# Work, Training and Life Stress in ITU World Olympic Distance Age-Group Championship Triathletes

**DOI:** 10.3390/sports11120233

**Published:** 2023-11-24

**Authors:** Veronica Vleck, Luís Miguel Massuça, Rodrigo de Moraes, João Henrique Falk Neto, Claudio Quagliarotti, Maria Francesca Piacentini

**Affiliations:** 1 CIPER, Faculdade de Motricidade Humana, Universidade de Lisboa, Cruz Quebrada, 1499-002 Lisbon, Portugal; 2 ICPOL, Instituto Superior de Ciências Policiais e Segurança Interna, 1300-663 Lisbon, Portugal; luis.massuca@gmail.com; 3 CIDEFES, Universidade Lusofóna, 1749-024 Lisbon, Portugal; rodrigodemoraes2@gmail.com; 4 Athlete Health Lab., Faculty of Kinesiology, Sport and Recreation, University of Alberta, Edmonton, AB T6G 2R3, Canada; falkneto@ualberta.ca; 5 Department of Movement, Human and Health Sciences, University of Rome ‘Foro Italico’, 00135 Rome, Italy; claudruns@hotmail.it (C.Q.); mariafrancesca.piacentini@uniroma4.it (M.F.P.)

**Keywords:** Life Stress, polarization, monitoring, training efficacy, amateur triathlon

## Abstract

We assessed the training, work and Life Stress demands of a mixed gender group of 48 top amateur short-distance triathletes using an online retrospective epidemiological survey and the Life Events Survey for Collegiate Athletes. On superficial inspection, these mainly masters athletes appeared to undergo all the types of training that are recommended for the aging athlete. However, there were significant scheduling differences between their weekday vs. their weekend training, suggesting that age-groupers’ outside sports commitments may affect their training efficacy. The triathletes claimed to periodize, to obtain feedback on and to modify their training plans when appropriate—and some evidence of this was obtained. Over the year preceding the ITU World Age-Group Championships, they averaged 53%, 33% and 14% of their combined swim, cycle and run training time, respectively, within intensity zones 1, 2 and 3. Although the triathletes specifically stated that their training was focused on preparation for the ITU World Age-Group Championships, the way that they modified their training in the month before the event suggested that this aim was not necessarily achieved. Sports-related stress accounted for most—42.0 ± 26.7%—of their total Life Stress over the preceding year (vs. 12.7 ± 18.6% for Relationship-, 31.3 ± 25.9% for Personal- and 14.0 ± 21.1% for Career-related Stress). It affected most athletes, and was overwhelmingly negative, when it related to failure to attain athletic goal(s), to injury and/or to illness.

## 1. Introduction

The sport of triathlon involves sequential swimming, cycling and running under conditions that differ from those of its component single-sport events [1]. The triathlon swim, cycle and run are linked, within the same event, by (- in the case of a multi-lap swim- a possible swim–run–swim transition that by us is termed T0), a swim–bike transition (T1) and a bike–run (T2) transition. According to which of a range of possible distances are involved, triathlon competitions can be categorized as being either “short-distance” or “long-distance” events. Both types of event are competed in at either the “elite” level or at the “age-group” level [2,3]. Entry into elite-level competition is dependent on the individual athlete’s national, regional and/or world ranking. In age-group competition, athletes compete against others who are within the same 5-year age band as themselves. Only short-distance triathlons, however, figure within the qualifying process for, and the actual, Olympic Games.

Millet, Bentley and Vleck [4] analyzed the evolution of the triathlon research literature from 1984 to 2006. They pointed out that the very fact of the triathlon becoming an Olympic sport had prompted researchers to investigate features (such as T2) that are specific to, and influence overall performance within, it. They also commented that coaches and athletes appeared to be benefiting from these studies, “first, because most of the leading scientists in the field have been athletes or coaches themselves… facilitating communication with their peers; second, (because), a good proportion of these studies have been conducted with national team athletes, and third, given the relative infancy of this sport and its multi-component nature, one might expect the empirical, field-based coaching knowledge to be more adaptable than in other endurance sports with a longer history and a more narrow range of skills”. The same authors also highlighted the fact that triathlon can be, and indeed has been, used as a model to investigate the effects of both cross-training practice and training mode-specific adaptations. It is perhaps both surprising and unfortunate, therefore, that over the intervening 16 years since [4] was published, few additional data have emerged about how triathletes train. Moreover, much of the related physiology literature focuses on elites. It is not necessarily easy to directly apply what data have since been published to the improvement of the training practice of age-group triathletes in particular. Such age-group athletes actually make up the majority of the participants in the sport [5].

When it became an Olympic sport, the focus of elite short-distance triathlon training changed from non-drafting- to draft-legal- competition. Draft-legal triathlons have very different physiological demands from those of amateur triathlons. The latter have remained non-drafting [2,3,6,7]. The following quote, from Sperlich, Treff and Boone [8], could just as well apply to elite and amateur triathlons: they “display strikingly different characteristics related to metabolic stress (i.e., magnitude of aerobic and anaerobic energy contribution), biomechanical loading (…), psychological challenges (…), environmental factors, competition features (duration, pacing, drafting and format) and also the timing and duration of the competitive season. Therefore, the performance defining factors are specific to the discipline and to the season and as such will strongly affect the training characteristics”.

Triathlon training can be characterized in terms of training intensity distribution (TID). This is the proportion of time that is spent within each of three training intensity zones [9], namely, zone 1, at or below the first ventilatory threshold (<VT_1_); zone 2, between the first and the second ventilatory thresholds (VT_1_-VT_2_); and zone 3, at or beyond the second ventilatory threshold (>VT_2_) [10]. A few studies of the training of highly performing elite, full-time, triathletes have been published (e.g., [11,12]). Such athletes appear, from these few data, to generally follow a polarized training model. They spend a high percentage of their training time within zone 1. They also spend greater percentages of their training time within zone 3 than they do within zone 2. This polarization may be combined with a pyramidal TID distribution, in which case a higher relative percentage of total training time is spent in zone 2. The actual percentages of training time that are spent in each zone will, of course, vary, depending on which part of the training year is involved. The problem is, as mentioned before, that these findings about elites essentially relate to “a different sport” within triathlon. They are also case studies. Case studies reflect personal coaching/athlete signatures rather than general or evidence-based practice. Not necessarily that much information can be gained from them as regards how to optimize *amateur* triathlon training practice. This is not least because the “off training stressors” of “full-time” (elite) and “part-time” (amateur) athletes will obviously differ. Such off training stressors can include training other than swimming, cycling and running, which themselves can considerably alter TID proportions [13]. There are also simply insufficient data in the current literature to allow for an informed judgement to be made on the extent to which the published training-related data for elite triathletes can be extrapolated to that of amateurs.

To date, the most detailed examination of triathlon training that relates its characteristics to *the extent of* ensuing maladaptation, in a *group* of short-distance specialists, that exists in the literature is *still* Vleck’s 1996 longitudinal prospective study of British National Squad athletes. The study lasted seven months and was published in full in [14]. Importantly, the data collection for the study predated the funding injection into British triathlon that led to more of its athletes being able to both focus on the global race circuit and to “go full-time”. As such, the research covered the preparation of amateur athletes for *non-drafting* National- and European Championship-level triathlon competition. This means that the training (and the TID) data that were obtained by the study likely more closely approximate those of the top age-groupers, as opposed to those of the professional triathletes, of today.

All of the subjects who took part in Vleck’s study were top-50 finishers at their National Olympic distance (OD) Championships, either within the year of, or within the year prior to, the study. The athletes’ (1.5/40/10 km) OD performance times are commensurate with, and still even perhaps slightly faster than, those that are currently achieved by (age-matched) top-level amateurs. The usual length of their competitive seasons and their training focus appear to be similar. That is, the training of both groups was built around National, Continental, (and then World) Championship level age-group competition. These facts underscore the potential interest of the data that were collected within Vleck’s prospective study to those who are looking to optimize age-group training. However, Vleck’s study purposely excluded athletes who were over 35 years old. Such “masters” triathletes are now of increasing research interest, for two reasons. Firstly, masters athletes account for the majority of amateur triathletes. Secondly, on superficial inspection, the multi-disciplinary endurance-based, high-intensity and resistance exercise training that is involved in preparing for a triathlon appears to comply with most, if not all, of the most recently published exercise guidelines for the aging athlete [15]. Applied research into the efficacy of training practice in age-group masters-level triathletes thus provides an opportunity to explore the appropriateness of the aforesaid guidelines. Unfortunately, no training *and* maladaptation data of an equivalent depth and study duration to that which Vleck [14] obtained for under-35-year-olds exist for older triathletes who are preparing for non-drafting, OD triathlon competition. It is unclear, therefore, to what extent the training practice of such masters triathletes may actually be optimal.

Fairly recently, Falk Neto et al. [16] prospectively assessed the training and maladaptation of nine recreational triathletes. They did so over the 6 weeks that led up to, and for 2 weeks after, an OD triathlon that was a key event of the athletes’ competitive season. Falk Neto et al. used the session rating of perceived exertion method to monitor their athletes’ daily training load. They also administered the Daily Analysis of Life Demands, the Training Distress Scale and the Alberta Swim Fatigue and Health questionnaires to the athletes every week. They found no discernable pattern in the athletes’ swim, bike and run training load within the five weeks leading up to the race. They also reported high variability in training load over the entire study duration. The triathletes spent an average of 47% of their training time in zone 1. More than half of their training, i.e., 25% and 28% percent, respectively, was spent in zones 2 and 3. In only 2 out of the 8 weeks of the study was a greater amount of training time spent in zone 1 than was spent in zones 2 and 3 combined. The authors concluded that, with their “large spikes in training load and a high overall training intensity”, their age-group sample had *not* apparently followed what is generally considered to be ideal endurance training practice [17]. Certainly, large spikes in training load have been linked to increased risk for injury and illness in triathletes. So, too, has combined, weighted, higher-intensity bike and run training [18,19]. However, although two out of the nine won their age-group, most of Falk Neto et al.’s subjects were neither particularly experienced, nor particularly successful, triathletes. The fact that their performance levels were heterogenous limits our capacity to make specific inferences about age-group triathlon training practice from their data. We do not know to what extent those athletes who compete at the *top* level of the amateur triathlon may exhibit similar (potential) “training mistakes”. Given that qualification for the World Age-Group Championships is performance-based, being on the start list for it could be used as an appropriate subject selection criterion for such individuals.

We do note that Falk Neto’s athletes did not manifest adverse effects from their apparent “departure” from general training guidelines. But, as the study was only 8 weeks long, this finding was not unexpected. The small number of study participants might also be why, out of all of those that were used, only the Alberta Swim Health Questionnaire flagged up symptoms of fatigue. The overall academic literature cannot be easily grouped into that which deals with elite vs. that which deals with age-group triathletes [14]. What it does say about the injury- and illness-related consequences, and the impact to general health, of triathlon training and racing was reviewed by [2,20,21], the consensus being that, as long as certain limits are respected, triathlon participation is fairly safe for the well-trained athlete [22]. However, said academic literature has barely examined the *psychological* effects of triathlon participation [23]. Emerging evidence also suggests that in amateurs, this effect is largely positive. Parsons-Smith et al. [24] observed, on the basis of the k-cluster of pre-performance scores, only 1.5% (out of a sample of 592) age-group triathletes to exhibit the “inverse Everest” profile—which is associated with elevated risk of psychopathology—on the Brunel Mood Scale. This 1.5% prevalence of the inverse Everest Profile in age-group triathletes is strikingly lower than the 5% prevalence of the general population. Even so, practically zero information exists in the academic literature on how the amateur triathlete’s mental and physical health are influenced by lifestyle and morbidity factors, or vice versa.

We again draw the reader’s attention to the fact that “age-groupers” are, by definition, not full-time triathletes. How the best age-groupers manage to fit training around their professional and other non-sports-related commitments is unclear. This issue is important because it will clearly have an impact on training quality. The relative levels of sports, relationship, career and personal stress that top age-groupers experience [25] are also insufficiently investigated. We know that people who participate in swimming, cycling and running can exhibit lower levels of psychological distress scores than those who walk or do no physical activity [26]. We also know that difficulty in balancing their training and other life commitments may lead recreational triathletes to experience high levels of negative stress [27]. However, we do not know what the major contributors to this negative stress are in this athlete group, which of these risk factors are potentially the most easily modifiable, nor how such risk factors potentially interact with each other. For example, mainly club-based training may mean that older triathletes experience less social loneliness. But, if the training that is provided by it is not triathlon-specific and does not take training in all the individual triathlon disciplines into account, club-based activity may not have a wholly positive impact on injury risk. We also know little of the degree to which triathlon training efficacy is monitored by or in age-groupers [2,14]. Nor has the extent to which top amateurs exhibit training flexibility, and respond to changes in their circumstances by taking positive action to minimize the risk of subsequent training maladaptation, been examined. Logically, such issues are of research interest.

Therefore, we analyzed the work, training and racing habits, and associated Life Stress, of top age-group triathletes who were both on the start line of, and indicated that the main goal that they had focused their training towards was, International Triathlon Union (ITU) World Age-Group Championship-level, OD competition. Both because this was an exploratory study and to increase subject numbers, we used a retrospective study design. Such a design is less sensitive than the longitudinal prospective one that was implemented by [14,16]. However, the training volume and intensity distribution were, in this case, examined across successive training blocks, rather than across successive weeks, of the entire year leading up to the 2013 ITU World Age-Group Championships. Where possible, we looked for divergence from accepted “training norms”. We also examined the data for the possible existence of/lack of relationships between potential “training”-related factors and failures. Our aim, in so doing, was to be able to suggest potential directions for future applied research into how to optimize training and maximize performance in age-group triathletes.

## 2. Materials and Methods

### 2.1. Subjects

Overall, 4348 athletes were registered to race in the 2013 ITU Triathlon World Elite and Age-Group Championships over the Team Relay, Sprint and Olympic Distance events. The event also included an Open race (i.e., one that did not require prior race performance-based qualification). Of the athletes who were on the Race Director’s contact list, 602 gave their written informed consent and participated in a mostly retrospective epidemiological survey. They did so after having received an emailed request to that effect. Each subject who requested it was emailed a signed copy of a confirmation of data confidentiality form. He or she was then sent an individualized link to an online training, injury, illness and stress-related questionnaire. The survey opened for completion three days after the 2013 ITU World OD Age-Group Championships. It closed one month after the event.

This paper reports data for those age-group triathletes who were on the start list for the ITU World Championships who (i) specifically stated that their training over the previous year was focused on preparation for OD competition and (ii) answered the section of the online survey that dealt with their training intensity distribution over the previous year *in full*. They accounted for 48 out of the 602 athletes who had provided written informed consent to participate.

### 2.2. Survey Content

Both the survey and the study were approved to proceed, before the race took place, by the Institutional Ethics Committee of Faculdade de Motricidade Humana, University of Lisbon. The survey covered the athlete’s medical history, sporting background, performance level, level of coaching support and training feedback, work commitments, the general structure of his/her training week, and his/her injury and illness history. Said questions were updated from, and built on the results of, previous research surveys that were conducted by the lead author. These investigations into triathlon training and maladaptation had been conducted successively over a period of approximately 30 years. They are listed within the bibliographies of [2,20,21]. The Life Events Stress for Collegiate Athletes (LESCA) [28] was included in the survey, with Professor Petrie’s permission. Brief details of both are provided below.

#### 2.2.1. Medical History

The athletes indicated whether they had personal/familial cardiovascular issues, a history of fainting/dizziness/loss of consciousness while/not while exercising, asthma/exercise-induced asthma, allergies and/or were suffering from serious illness/condition(s) that could affect the ability to exercise. They could also indicate which medication(s) they were on (if any) and why, if they chose to do so.

#### 2.2.2. Sporting Background/Performance

The age-groupers indicated their years of training and racing experience in both triathlon and its component sports. Usual times over the previous year and over the most common triathlon race distances were also provided. The athletes differentiated between whether such times had been recorded for individual swim, cycle or run time trials, or within triathlon competition. The athletes confirmed which event(s) and category they raced in at the ITU World Championships. They provided self-assessments both of their individual prowess in each of triathlon’s component disciplines (including both the swim–bike and the bike–run transitions) and of their current training status. These data were used, in conjunction with data from other sections of the survey, to assess the extent to which self-identified/potential weakness(es) (Section 2.2.3, Section 2.2.4 and Section 2.2.5) were being identified or not. They were also used to identify whether active action was subsequently being taken by the athletes to address their weaknesses.

#### 2.2.3. Support and Commitments

The extent to which athletes trained with single-sport athletes and clubs, the type of coaching that they received and the extent to which their technique in each of the sections of a triathlon was usually analyzed were reported. We also asked about whether the athletes were in part/full time work/study or self-employed, about their average weekly number of working hours and whether (such) work was full-time or shift-based. Details (with session times) of how each athlete fitted swim, bike, run and weight training sessions around his/her work and other commitments were obtained. So, too, was information on the extent to which the athletes considered their training to be polarized, implemented goal setting, and modified their training as a result of failure(s) to attain their goals, injury and/or illness.

#### 2.2.4. Training Duration, Frequency and Intensity Distribution

Moreover, for each of the so-called training phases (i.e., the Endurance Base-EB, Pre-Competition-PC, Competition-C, Taper-T and Off-season-Off) of their training year, the athletes self-reported their usual weekly training durations and frequencies for swimming, swim–cycle training, cycling, cycle–run training, running, weight training and other sport/exercise modes. Weekly training durations and frequencies were also obtained for the types of sessions that Vleck’s studies [14,18,20,29,30] have previously specifically examined with regards to their potential associated overuse injury risk (i.e., for swim–bike transition, “long bike”, “hill reps bike”, “speed work bike”, bike–run transition, “other bike”, “long run”, “hill reps run”, “speed work run” and “other run” sessions). For swimming, cycling and running training only, average weekly training times within each of five exercise intensity levels, of which level five was the highest [14,18], were also recorded for each training block. Details of how each such exercise intensity level related to various physiological markers, to the Borg CR-10 scale of perceived exertion and to the “talk scale” were provided to the athletes. The triathletes were also given examples of both the types of work to rest ratios that exercising within each intensity level would involve for swimming, cycling and running, and example training sessions for them (Table 1).

Said information was based, with permission, on previously published United States Olympic Committee athlete training guidelines for swimming, cycling and running. These guidelines are detailed in [14]. They had been cross-checked and their suitability for triathletes agreed to by three British National Triathlon Squad coaches before they were first used within Vleck’s longitudinal study [14]. Training intensity levels 1 and 2 together, level 3, and levels 4 and 5 together were, for the purposes of this paper, classed as being synonymous with training intensity zone 1 (z1), with zone 2 (z2) and with zone 3 (z3), respectively. As such, they were thus considered to broadly correspond to the following exercise intensities: (i) <VT_1_; (ii) VT_1_–VT_2_; and (iii) >VT_2_, respectively. All the training duration-related data that were collected from the athletes were estimated to the nearest half hour. This was the case, whatever the specific session type that the information related to.

The athletes who took part in the survey originated from various countries around the globe. The ITU World Age-Group OD Championships took place in late September in London, England. This meant that the event did not necessarily fall within the competitive phase of the racing season of an individual athlete’s country of residence. Therefore, each competitor also reported whether the month leading up to the World Championships fell within the Endurance-Base, Pre-Competition, Competition, Taper or Off-Season phase of his/her training year. They also reported whether and how their amount of recovery and number of races within said month differed from what was customary for them for that type of training phase. They gave the same information regarding their weekly training frequencies, total weekly training times and individual training session durations for each swim, cycle and run intensity level.

#### 2.2.5. Life Stress

The athletes also completed “The Life Events Survey for Collegiate Athletes”, or LESCA [28]. This survey was used with the author’s permission. The LESCA has previously been shown to possess good content validity. It also provides a stable measure of Life Stress. Scores on the LESCA have also been shown to be a better predictor of athletic injury than those that are obtained from the Social and Athletic Readjustment Rating Scale [28]. The LESCA involves an 8-point Likert scale. This allows individuals to not only rate the degree of stress (from 1 to 4, that is, from minor to major), but also the type of stress impact (i.e., beneficial/positive or detrimental/negative) that has been experienced by them over the previous year.

Three different Life Stress scores were obtained from the athletes’ ratings on the LESCA. Negative Life Stress and Positive Life Stress scores were derived by summing the impact scores of those events that were rated as undesirable (negative) and desirable (positive), respectively. A Total Life Stress score was obtained by adding the absolute values of the negative and positive scores. Total, positive and negative stress values were also calculated for the following sub-components of Life Stress variables: (i) Sport; (ii) Relationship; (iii) Career; and (iv) Personal Stress. These were derived from the scores from questions whose original number in Petrie’s LESCA paper were as follows (i) for Sport Stress: 15–16, 24, 33–35, 42, 44–57, 63–64 and 67; (ii) for Relationship Stress: 1, 8–10, 12–14, 17–18, 29–31, 39–40 and 60; (iii) for Career Stress: 11, 19, 21, 23, 25, 32, 58 and 61–62; and (iv) for Personal Stress: 2–7, 20, 27–28, 36, 38, 40–41, 43 and 66–68.

#### 2.2.6. Injury and Illness

Finally, injury and illness data both for the year and for the month prior to completion of the survey were obtained. This was in addition to the reporting of injury and/or illness that occurred *at* the World Championships. The definitions and methods of data collection for the online survey-based injury-related data were those that have been consistently implemented by Vleck [14,18,20,29,30], as reviewed by Vleck and Hoeden [20]. They included details of the anatomical location, the severity and the recurrence of both overuse and traumatic injury. The survey explicitly stated that “here an injury is defined as any musculoskeletal problem that caused you to stop training for at least one day, reduce mileage, take medicine or seek medical aid. Traumatic injuries are those caused by hazard encounters such as hitting a car or falling off your bike and overuse injuries are those that you would consider to have been caused by repetitive strain. The following definitions of severity also apply, both for injury and for illness: Minor: ‘1–4 days lost’, Moderate: ‘5–14 days lost’, Severe: ‘15 or more days lost’, Out of season: resulted in your entire season being affected, regardless of where the injury or illness occurred in the season. Recurring injury: an injury that occurs more than once, with an interval of at least 7 days between successive recurrences.” Where, when and why the injury or illness was considered by the athlete to occur were also noted. Details of the degree, timing and type of clinical/non-clinical support for injury and/or illness that was sought/obtained by each individual, both during and after the ITU World Championships, were collected. Medical tent/team-based clinical injury and illness data were also collected over the duration of the ITU World Championships. None of the athletes who featured in this particular analysis presented for such clinical assistance. Therefore, no such clinically diagnosed on-site injury/illness data were available for this specific paper.

During the data collection period and for each of the above-named individual survey sections, the athletes were encouraged to add personal comments/explanations of their replies. They did so extensively. The athletes were also encouraged to request clarification from the lead author where necessary.

### 2.3. Statistical Analysis

The level of coaching specificity that the athletes received was compared between the three triathlon disciplines using the Chi-squared test. All the training data that were obtained were scatter-plotted. In almost no cases were the athletes’ training data normally distributed. Levene’s test for homogeneity of variance was conducted before any demographic data were compared.

With regards to the analysis of the athletes’ “work sports balance”, (i) the Wilcoxon signed-rank test was used to study the differences between weekday vs. weekend training, and (ii) the Friedman two-way analysis of variance by ranks was used to examine the differences between swim vs. bike vs. run vs. weights vs. other, the differences in the weekly training frequency and training time by exercise mode (i.e., swim vs. swim–bike vs. bike vs. bike–run vs. run vs. weights vs. other) and by training block (i.e., EB vs. PC vs. C vs. T vs. Off) and the differences in the (frequency and training time per mode related) details of the athletes’ swim, bike and run training sessions. Where significant differences were revealed by the Friedman test, a post hoc test with Bonferroni correction was then used to identify which specific training data pairs differed from each other.

In order to identify whether the training durations within each of the five exercise intensity zones varied significantly from each other during each phase of the periodization, Friedman’s two-way analysis of variance by rank*s* and post hoc tests with Bonferroni corrections were again used. The same tests were used to check whether there were significant differences in time, or in percentage of time, in each training intensity, between the five phases of the training year (i.e., EB, PC, C, T and Off).

Student’s *t*-test for independent variables was also used to check for significant differences between the time spent at each level of training intensity, for each type of training block, of the 10 fastest and the 10 slowest performers in the World Age-Group Championships in the group.

The ‘Statistics Package for the Social Sciences’ (SPSS, High Wycombe, UK), version 28.0, was used throughout the analyses. We set the 95% confidence limit as the level of statistical significance. Given the large number of analyses that were involved in the preparation of this paper, and in the interests of decluttering, some statistical data have been omitted from its training data-related Tables and Figures. In such cases, the data that *are* provided in this paper are given in exactly the same format (i.e., as means ± standard deviation SD) as they have been provided in the majority of the triathlon literature. This allows for direct comparisons to be made between the data in this paper and those from the previously published studies that employed an identical data collection methodology (as reported within [14,29,30]). The relevant median, interquartile ratio, mean rank data, etc. are available from the first author.

## 3. Results

### 3.1. Subject Characteristics

Forty-eight age-groupers, competing at ITU World OD Age-Group Championship level, fulfilled the subject criterion for this study. These 21 males and 27 females were spread across the age-groups from 20–24 up to 65–69 years old (Table 2). Overall, 68.7% of the study participants were 35 years of age or older.

The athletes possessed an average training and (in brackets) racing background of (mean ± SD) 10.2 ± 6.2 (9.0 ± 6.2) years in triathlon, 13.3 ± 7.2 (9.6 ± 7.9) years in swimming, 10.6 ± 5.8 (6.5 ± 5.9) years in cycling, 18.9 ± 8.0 (16.2 ± 7.5) years in athletics and 16.0 ± 7.4 (13.7 ± 7.2) years in other sports. They placed from 2nd to 122th (averaging 36.5 ± 26.9 th) within their respective age-groups at the 2013 ITU World Age-Group Championships. Their average times for Olympic distance triathlon competition were 2:13:25 ± 0:16:07 (1:58:55–3:00:00) hh:mm:ss for the males and 2:32:57 ± 18:26:46 (2:11:56–3:18:00) hh:mm:ss for the females. Detailed (single-sport time trial, as well as triathlon-specific) performance-related data for each age-group that was represented in this study are also available from the first author on request. So are self-assessed rankings of each of the individual athlete’s relative swim, swim–bike transition, bike, bike–run transition and running ability within triathlon competition.

### 3.2. Training Support

Almost half of the athletes in the group (47.9%) were regularly training with single-sport specialists. Those who sometimes did so and those who never did so accounted for 31.3% and 20.8%, respectively, of these top age-groupers. The type of coaching that the athletes received differed between the three triathlon disciplines. More often than not, the athletes received club-based coaching (Table 3). More athletes were swimming club or triathlon club members (50.0% and 58.3%) than were members of cycling or athletics clubs (27.1% and 25.0%), other clubs (6.3%) or not in any sports club (10.4%). In only approximately a third of cases, whatever the exercise mode, did the athlete consider his/her club-based coaching to be triathlon-specific. Of the athletes who were coached, other than self-coached, and whose coaching was geared towards triathlon: 27.1% had the same coach overseeing their training for all three triathlon disciplines “all the time”; 20.1% had it for “most of the time”; 12.5% had it “sometimes”; and 25.0% “never” received that level of support.

Almost all (95.8%) of the group indicated that they had “set goals” for the 2013 season. In all such cases, these goals were specifically stated to have been to beat a specific competition time, to qualify for the World Championships, to compete in the World Championships or a combination of the aforesaid goals. Seventy-eight percent of individuals also indicated that they had a periodized training plan for the season. This plan had been worked out either by themselves or together with their coach, and was based around their aforementioned goals.

### 3.3. Life–Sports Balance and Load

#### 3.3.1. Balance between Training and outside Sports Commitments

For the majority (62.5%) of the athletes, their training plan was built around working at a full-time job. Just over a third (31.3%) of the group were part-time workers, and 8.3% were self-employed. Half of the triathletes worked fixed hours, while 12.0% did shift work. On top of working/studying 7.6 ± 2.5 h per day, these top age-groupers trained 1.7 ± 0.5 (min–max, 0.57–3.0) times every day. They did so irrespective of whether the day in question was a weekday or fell over a weekend. We did not assess time spent looking after family members (and children, in particular) within the survey. Nonetheless, some athletes commented that had it not been possible to share such responsibilities with a partner, they would potentially have had a detrimental impact on their training. The athletes’ training schedules were fairly variable, but were clearly influenced by their weekday commitments. Both the proportion of their total number of weekly training sessions that were planned within it and the type and the timing of said sessions differed between weekday- and weekend-based training (Table 4).

Over 88% of the athletes’ 3.1 ± 1.1 scheduled weekly swim training sessions took place within the working week. A total of 52% of them were fitted in before 0900 h, and 28.3% of them took place between 1800 and 2200 h. That is, the majority of the swims were performed before or after what are usually considered to be normal working hours. During weekends, no one reported swimming within the 1800–2200 h slot. The situation as regards the usual timing of the athletes’ 3.4 ± 1.2 weekly bike training sessions was less clear-cut. Over half of them were performed during the week. Most (45.8%) of the athletes did none of their within-week bike training sessions before 9 am. Less than a third (27.1%) did between a third and half of such sessions before 9 am. Only 12.5% did all of their weekday bike training before 9 am. Those who did none, 1/3–1/2 and all of their “work week” bike sessions between 0900 h and midday accounted for 41.7%, 16.8% and 10.4% of the group, respectively. As for the “lunchtime” slot of between 1200 and 1500 h, 75% of the group did none and 8.4% did 1/3–1/2 of their weekday bike sessions in it. Between 1500 and 1800 h, 18.9% of the athletes did 1/3–1/2 of their bike sessions, but 66.7% of them did none of them. In total, 54 %, 41.9% and 10.4% of athletes, respectively, did none, 1/3–1/2 and 25% of their weekday sessions after normal working hours, i.e., between 1800 and 2200 h. Within weekends, the way the athletes’ cycle training was scheduled changed. A far larger proportion of this training was performed before midday than occurred during the week. As for running training, the athletes’ normal training plan involved 3.6 ± 1.6 such sessions per week. Of these runs, 2.2 ± 1.1 were carried out over the Monday–Friday period. A further 1.3 ± 0.8 runs were performed over the weekend. Of the five weekday run “time-slots” that we assessed, the one that accounted for the largest proportion of scheduled runs was the one before 0900 h. In fact, 18.8% and 14.7% of athletes did all or between 40% and 66%, respectively, of their weekday runs before 9 am. Overall, 25% of the group did between 25% and all of such runs between 1200 and 1500 h, 20.9% did between a third and all of them between 1500 and 1800 h, and 38.9% did between a quarter and all of them between 1800 and 2200 h. Then, again, the way that scheduling of this training occurred changed during the weekends. Finally, the athletes’ weight training sessions, of which 1.1 ± 1.4 were normally scheduled per week, were also mostly performed during the working week. This swimming, biking, running and weight training was not all the training that the athletes were doing, however. The triathletes reported themselves to also be normally doing 0.3 ± 0.7 weekly training sessions in other sports.

#### 3.3.2. Training and Racing Load

The athletes competed in an average of 3.2 ± 4.4 (range 25) 5 km runs, 1.0 ± 1.2 (range 5) 10 km runs, 0.7 ± 1.0 (range 4) half marathons and 0.2 ± 0.5 (range 2) marathons over the year leading up to the World Championships. They also took part in 1.4 ± 2.0 (range 9) 10-mile cycle time-trials and 0.9 ± 1.9 (range 10) 40 km cycle time trials. On average, they competed in 2.3 ± 2.2 (range 9) Sprint distance triathlons, 3.5 ± 1.7 (range 8) Olympic distance triathlons, 0.1 ± 02 (range 1) middle-distance triathlons, 0.1 ± 0.4 (range 1) Half Ironman distance triathlons and no Ironman distance triathlons. The time that the athletes estimated that they had spent racing over the same year was calculated as 17.5 ± 7.0 h on average (min–max, 5.5–35.5). The latter value was arrived at when what were presumably rogue values that were given by 4 athletes (of 532, 600–650, 550–600 and 20 h racing per week over 12 months) were ignored.

**Table 5:** Weekly training frequencies and total training time in h (mean, SD), by training phase.

		EB	PC	C	T	Off
Swim (S)	Frequency	3.6 ± 1.2	3.9 ± 1.1	3.9 ± 0.9	3.2 ± 0.9	2.9 ± 1.1
	Total time (h)	6.6 ± 3.7	7.0 ± 3.6	6.7 ± 3.2	4.9 ± 2.5	4.8 ± 3.1
Swim–Bike (SB)	Frequency	1.2 ± 0.5	1.6 ± 0.9	1.6 ± 0.6	1.4 ± 0.6	1.0 ± 0.2
	Total time (h)	1.2 ± 1.3	1.7 ± 1.9	1.9 ± 1.7	1.2 ± 1.1	1.3 ± 1.5
Bike (B)	Frequency	3.6 ± 0.9	4.0 ± 1.1	4.1 ± 1.1	3.4 ± 1.0	2.9 ± 1.3
	Total time (h)	9.9 ± 4.6	10.5 ± 5.0	10.1 ± 4.8	6.8 ± 3.7	6.8 ± 4.4
Bike–Run (BR)	Frequency	1.7 ± 0.8	2.2 ± 0.9	2.3 ± 0.8	1.8 ± 0.8	1.2 ± 0.5
	Total time (h)	2.4 ± 2.3	3.4 ± 3.1	3.6 ± 2.9	2.3 ± 2.2	0.9 ± 0.8
Run (R)	Frequency	4.2 ± 1.1	4.1 ± 1.1	4.1 ± 1.0	3.5 ± 1.1	3.6 ± 1.1
	Total time (h)	7.1 ± 3.2	7.0 ± 3.0	6.5 ± 2.9	4.8 ± 2.5	5.8 ± 2.8
Weights (W)	Frequency	2.4 ± 1.1	2.1 ± 1.1	4.7 ± 0.9	1.3 ± 0.6	2.1 ± 1.3
	Total time (h)	3.0 ± 2.4	2.6 ± 2.2	1.9 ± 1.5	1.5 ± 1.1	2.6 ± 2.3
Other (O)	Frequency	1.9 ± 1.3	1.6 ± 1.3	1.5 ± 1.3	1.4 ± 1.2	2.4 ± 1.6
	Total time (h)	2.4 ± 2.9	1.7 ± 1.8	1.5 ± 1.7	1.4 ± 1.7	2.8 ± 2.8

Key: EB, Endurance Base; PC, Pre-Competition; C, Competition; T, Taper; Off, Off-Season; freq, frequency.

Statistics related footnote to Table 5 (Friedman’s test)^a^: (i) Differences in total training time—h-per mode (i.e., swim vs. swim–bike vs. bike vs. bike–run vs. run vs. weights vs. other): EB, *X*^2^(6) = 186.583, *p* < 0.001, N = 48; PC, *X*^2^(6) = 195.227, *p* < 0.001, N = 48; C, *X*^2^(6) = 195.659, *p* < 0.001, N = 48; T, *X*^2^(6) = 192.794, *p* < 0.001, N = 48; Off, *X*^2^(6) = 143.654, *p* < 0.001, N = 48; (ii) Differences in total training time—h-per training block (i.e., EB vs. PC vs. C vs. T vs. Off): Swim, *X*^2^(4) = 75.129, *p* < 0.001, N = 48; Swim–Bike, *X*^2^(4) = 37.409, *p* < 0.001, N = 48; Bike, *X*^2^(4) = 79.030, *p* < 0.001, N = 48; Bike–Run, *X*^2^(4) = 71.583, *p* < 0.001, N = 48; Run, *X*^2^(4) = 75.362, *p* < 0.001, N = 48; Weights, *X*^2^(4) = 65.685, *p* < 0.001, N = 48; Others, *X*^2^(4) = 39.307, *p* < 0.001, N = 48; (iii) Differences in frequency: EB, *X*^2^(6) = 186.583, *p* < 0.001, N = 48; PC, *X*^2^(6) = 195.227, *p* < 0.001, N = 48; C, *X*^2^(6) = 195.659, *p* < 0.001, N = 48; T, *X*^2^(6) = 192.794, *p* < 0.001, N = 48; Off, *X*^2^(6) = 143.654, *p* < 0.001, N = 48. Post hoc tests, EB vs. C, for time: *** BR; for freq: *** BR, W; * SB; for EB vs. T, for time: *** S, SB, B, R; for freq: *** SB, BR; for EB vs. Off, for time: *** S, B, * BR, R; for PC vs. T, for time: *** S, B, R, * BR; for freq: *** SB; for PC vs. Off, for time: *** S, B, BR, R, for freq: *** BR, W; for C vs. T, for time: ** S, B, BR, R; for C vs. Off, for time: *** S, B, BR, * SB; for freq: *** BR, * R, for freq:, *** W; for T vs. Off, for time: *** R; for freq: *** BR, * W. *** *p* < 0.01, ** *p* < 0.02, *p* < 0.05. ^a^ Significance values were adjusted by Bonferroni correction.

What these top OD age-groupers considered to be their own normal training, per 7-day week, is presented in Table 5 (above). A more detailed breakdown of their training frequency data is shown in Table 6. The aforesaid data are expressed in terms of swim, bike and run “speed”, “long”, “hill rep” sessions, etc. (as per [15,29,30]). Details of the athletes’ weight training are also provided in the same Table. The male and female training-related data are not reported separately because a prior analysis of 124 (Sprint, OD and team relay) age-group competitors who were competing at the same ITU World Championships [31] did not reveal any notable differences in training practice between the two genders.

Statistics related footnote to Table 6 (Friedman’s test, note that not all the athletes provided these data in full) ^a^: Differences between time used in training modes during training blocks, i.e., (i) EB, X2(9) = 115.666, *p* < 0.001, N = 26; PC, X2(9) = 89.839, *p* < 0.001, N = 26; C, X2(9) = 78.428, *p* < 0.001, N = 26; T, X2(9) = 45.654, *p* < 0.001, N = 25; Off, X2(9) = 75.545, *p* < 0.001, N = 26; and (ii) frequency: EB, X2(10) = 85.772, *p* < 0.001, N = 33; PC, X2(10) = 58.160, *p* < 0.001, N = 32; C, X2(10) = 63.225, *p* < 0.001, N = 31; T, X2(10) = 64.605, *p* < 0.001, N = 32; Off, X2(10) = 78.405, *p* < 0.001, N = 31. Post hoc tests, EB vs. PC: for freq: *** SWB, * for field SWB, for time: * both SWB; for EB vs. C, for freq: *** for SWB, ** T2; for time: *** T2; for EB vs. T, for freq: *** LB, LR; ** HRR; for time: *** LB, LR; for EB vs. Off, for both time and freq: *** LB; for PC vs. T, for freq: *** LB, HRB, LR; for time: *** LB, HRB, both SWB, LR, SWR; for PC vs. off, for time: *** LB, HRB, both SWB, T2, SWR; for freq: *** field SWB, T2, SWR; for PC vs. Off, for time: *** LB, HRB, SWR, * LR; for freq: *** LB, HRB; for C vs. Off, for time: *** both SWB, SWR; ** T1; for freq *** field SWB, T2, SWR, ** HRB; for T vs. Off, for time, *** LR; * T2; for freq *** field SWB, T2, ** LR. ***, *p* < 0.01, ** *p* < 0.02, *p* < 0.05. ^a^ Significance values were adjusted by Bonferroni correction.

#### 3.3.3. Changes in Training Intensity across the Training Year

**Figure 1:**
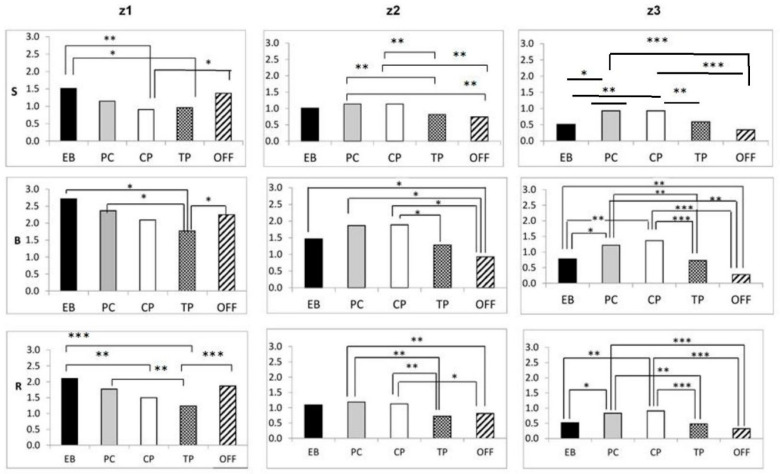
Average weekly training time (h) in each intensity zone, by discipline, by training phase. Key: EB, Endurance Base phase; PC, Pre-Competition phase; CP, Competition phase; TP, Taper phase; Off, Off-Season; S, swim; B, bike; R, run; z1, training intensity zone 1; z2, training intensity zone 2; z3, training intensity zone 3; * *p* < 0.05, ** *p* < 0.02, *** *p* < 0.01.

**Figure 2:**
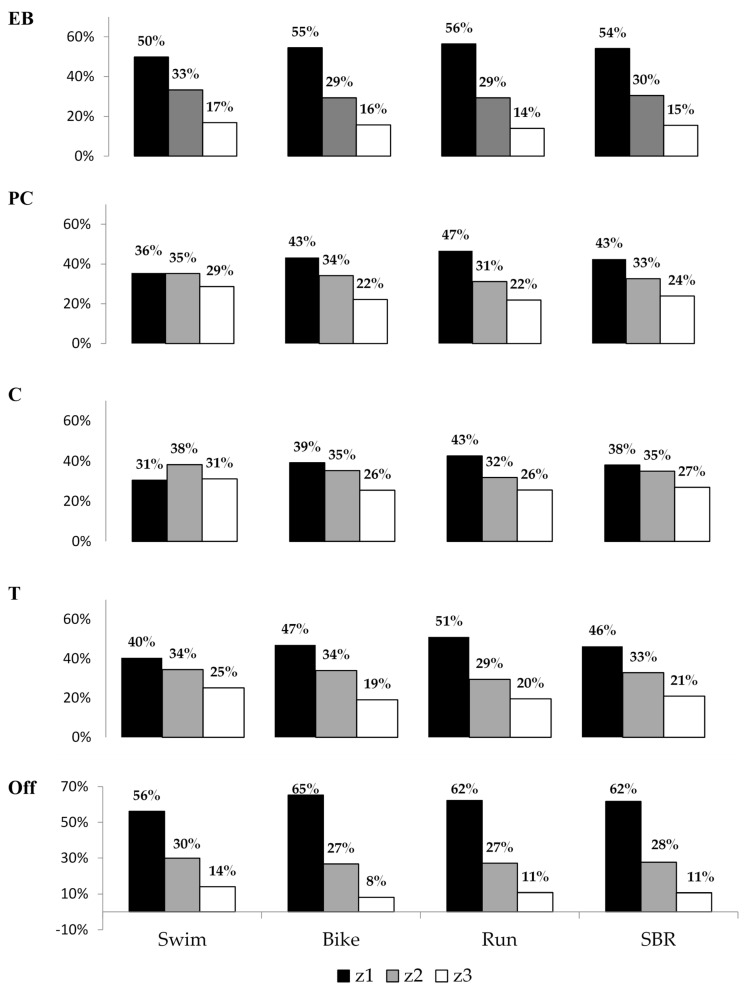
Percentage of training time spent in exercise intensity zones z1, z2 and z3, both overall and in swimming, cycling and running, within the Endurance-Base (EB), Pre-Competition (PC), Competition (C), Taper (T) and Off-Season (Off) phases of the year leading up to the World Championships. Key: SBR Combined swim, bike and run training.

The overall average weekly total (swim, bike and run) training time that was spent within each of the three training intensity zones z1–3 changed with the training phase (z1, *F*(4,239) = 3.478, *p* = 0.009; z2, *F*(4,239) = 9.222, *p* < 0.001; z3, *F*(4,239) = 16.163, *p* < 0.001) (Table 7 and Figure 1 and Figure 2). Within the Endurance-Base, Pre-Competitive, Taper and Off-Season phases, the training time that was spent in z1 exceeded that spent within z2 and z3. Within the Competition phase, z2 was the training zone in which the highest proportion of swim training time was spent. Most of the triathletes’ training time was spent cycling, regardless of what training macrocycle type or intensity zone they were in.

The athletes’ low-intensity (z1) swim or cycle training time did not vary significantly across training phases. This was not the case as regards their time spent doing higher-intensity work (i.e., swimming-z2, *F*(4,239) = 16.506, *p* < 0.001; swimming-z3, *F*(4,239) = 7.367, *p* < 0.001; cycling-z2, *F*(4,239) = 12.650, *p* < 0.001; cycling-z3, *F*(4,239) = 13.585, *p* < 0.001). Run z1, z2 and z3 training time did differ with training phase (z1, *F*(4,239) = 4.710, *p* = 0.001; z2, *F*(4,239) = 3.166, *p* = 0.015; z3, *F*(4,239) = 8.618, *p* < 0.001).

Training phase-based differences in the time that was spent exercising within each intensity zone were seen (i) for swimming, within each of the Endurance-Base, Taper and Off-Season periods; (ii) for cycling, within all five training cycles; and (iii) for running, within every phase, apart from within the Competition phase. The total swim, bike and run (SBR) training time that was spent within z1, z2 and z3 also differed within all the assessed training phases, apart from the Competition phase. Examples of this include (i) for the Endurance-Base phase, between z1 and z3 (*p* < 0.001), between z1 and z2 (*p* = 0.015) and between z2 and z3 (*p* < 0.001); (ii) for the Pre-Competition phase, between z1 and z3 (*p* < 0.001) and between z2 and z3 (*p* < 0.001); (iii) for the Competition phase, between z2 and z3 (*p* = 0.021); (iv) for Taper, between z1 and z3 (*p* < 0.001) and between z2 and z3 (*p* = 0.006); and (v) for the Off-Season phase, between z1 and z3 (*p* < 0.001) and between z1 and z2 (*p* = 0.001), as well as between z2 and z3 (*p* = 0.001).

From the data that the athletes themselves provided, it appears that the average percentage of their combined weekly swim, cycle and run training time that they normally spent within z1, z2 and z3, respectively, was 53%, 33% and 14%, respectively (Table 6, Figure 2). In no individual macrocycle or discipline did the average proportion of reported swim, cycle and run training that the athletes spent in z1 exceed 56%. The actual percentage of training time that was spent in z1 steadily dropped from the Endurance-Base phase through the Pre-Competition to the Competition periods. Training time within z1 was higher within the Taper and Off–Season phases than it was in other phases. Conversely, the relative proportion of training time that was spent in z3 generally progressively increased over the Endurance-Base to the Pre-Competition through to the Competition periods. It only did not do so when Taper work—which would normally immediately precede important races—was being performed. The training time that was spent within z2 varied by a maximum of 8% over the same periods.

We also compared the training of the fastest 10 and the slowest 10 triathletes in our subject group of 48. No significant differences were seen between the two sub-groups in either changes in training intensity across the training year or in TID. The data are not shown here. We note that the way the study participants’ finishing positions were spread across their age-group field was not perfectly matched between the different age-groups that featured in this study. This, our sample size and the fact that the number of athletes within the competition varied between the individual age-groups that featured in this study meant that we were unable to adjust the analysis for the fact that triathlon performance changes with age.

#### 3.3.4. Training Feedback and Adjustment

Most of the group (70.8%) indicated that they were receiving feedback on the aforesaid exercise training from a coach. This is a far larger proportion of the subject group than those who were receiving feedback from a physiotherapist (35.4%), from a general (medical) practitioner (4.2%), from a sports medicine doctor (8.3%), from lab testing (12.5%), from training camps (16.7%), from an online training diary (20.8%), from their heart rate monitor (47.9%), from the academic literature (16.3%), from the world wide web (39.6%) or from other sources (12.5%).

The regularity with which feedback on their swim, bike, run, swim–bike transition or bike–run transition technique was received, however, varied widely among individuals. Some individuals obtained such feedback after almost every training session, while others almost never obtained it. Technique feedback was consistently most often obtained for swimming and running. It was given less regularly for cycling, almost never for the bike–run transition and even less regularly for swim–bike transition training. There were also only low, non-significant correlations between the individual athlete’s self–assessment of their relative ability in swimming, the swim–bike transition, cycling, the bike–run transition and running and the regularity with which they received technique-related feedback for it.

#### 3.3.5. Changes from Normal Training in the Immediate Lead-Up to Worlds

**Figure 3:**
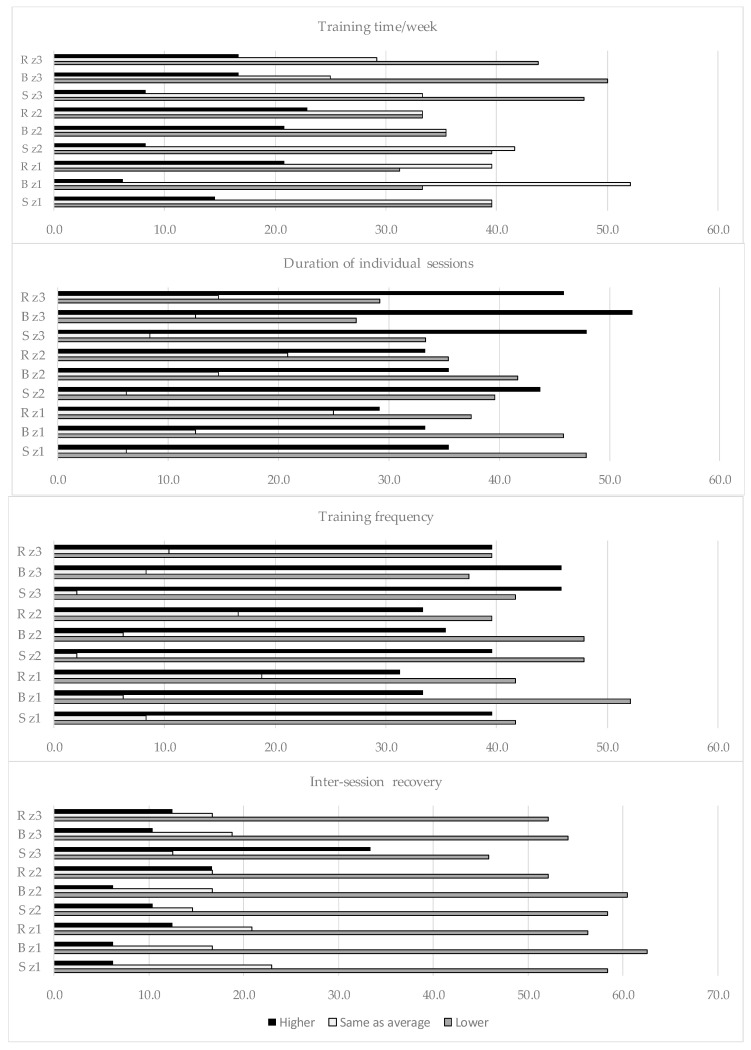
Proportion of the athletes who did/did not modify their training in the month leading up to the ITU Age-Group World Championships from that which was customary for them for the training phase within which it fell. Key: S, swim; B, bike; R, run; z1, zone 1; z2, zone 2; z3, zone 3.

The 48 athletes who took part in this study represented different countries and continents. None of the athletes indicated that these World Championships fell within the competition period of their training year. Only 6.3% of the subjects reported that the event fell within their Pre-Competition period. The World Championships fell within the Endurance Base period and within the Pre-Competition period in 45.8% and 47.9% of the group, respectively. How the athletes considered themselves to have altered their training over the month leading up to the event from what they considered to be their normal training for that period of the training year is illustrated in Figure 3.

Neither these changes nor their potential cause were particularly clear-cut. What stood out, however, was that, apart from as regards weekly training time, those athletes who did not change their training from what they themselves had recorded as normal for themselves appeared to be in the minority. Most of the athletes deemed their levels of inter-training session recovery to have been lower than normal within the month leading up to the World Age-Group Championships. However, a similar proportion of the group considered themselves to have lowered their training frequency in the higher run and bike intensity levels as deemed themselves to have increased it. Although more athletes than not increased their session duration in these same levels, the majority of the group indicated their total overall training volume in the month leading up to Worlds to have been less than customary. As for the number of races that the athletes competed in within the month leading up to Worlds, this was less than normal in 47.9% of the group, the same as normal in 45.8% of the group and more than normal in 6.3% of the group. Overall recovery times between sessions were thought to be less than, the same as and more than normal in 10.4%, 52.1% and 37.5% of the athletes, respectively.

This training modification was presumably at least partly schedule-related. It was performed in addition to the updating of goals and subsequent training that was implemented “at the end of each macrocycle”, “after each major race” and “after illness, injury and/or unexplained performance decrement” by 25.0%, 47.9% and 37.5% of the athletes, respectively. We note, however, that 22.9% of the group stated that they had not updated their goals and subsequent training from what had been set at the onset of the season, at any point within that season, when they finished the survey.

### 3.4. Health Status

Those who reported themselves to be affected by injury over the preceding year accounted for 16.7%, and over the month prior to survey completion, for 29.2% of the group. Overall, 12.5% of the athletes were injured at the World Championships. Injury prevalence at the exact moment of survey completion was 33.3%. A further 2.1% of the group were, at that point, as yet unsure as to whether they were injured or not. The details of these injuries shall be reported in a separate paper. Overall, the proportions of the group who were ill over the month prior to Worlds, and within the event, were 16.7% and 4%, respectively. All the illness cases that occurred within the month leading up to Worlds were related, by the athletes, to cold/virus/bronchitis, “a bug” and/or to flu-like symptoms. One athlete also contracted food poisoning. One had a concurrent ear/eye infection. At the point of survey completion, 4.2% of the group were ill. On the same occasion, i.e., inside one month of racing in the ITU World OD Age-group Championships, more of the athletes (39.6%) considered their state of training to be “good” than anything else. This compares to the 4.2% of the study participants who thought their training status to be very, very good; 18.8% who thought it to be very good; 20.8% who considered it neither good nor poor; and 16.7% who reported it as poor.

However, 22.9% of the group had competed while knowing—and 6.3% while unsure—that they had a family history of cardiovascular disease. The proportion of our subject group who had raced the World Championships with current cardiovascular issues or breathing difficulties was 10.4% (Table 8). In total, 9 out of the 48 (i.e., 18.8%) indicated themselves to have previously suffered or be suffering from a serious illness or condition that could affect their ability to exercise. In three individuals (6.3%), this limitation was at least partly due to their having suffered (training-related) traumatic injury. Moreover, 39.6% and 2.1% of the athletes reported themselves as suffering or possibly suffering from allergies, respectively. These allergies were mostly attributed to food/stings/dust/animals/selected medications. In one case, the cause of allergy was unknown to the individual, despite it previously having caused anaphylactic shock.

### 3.5. Stress Levels

**Figure 4:**
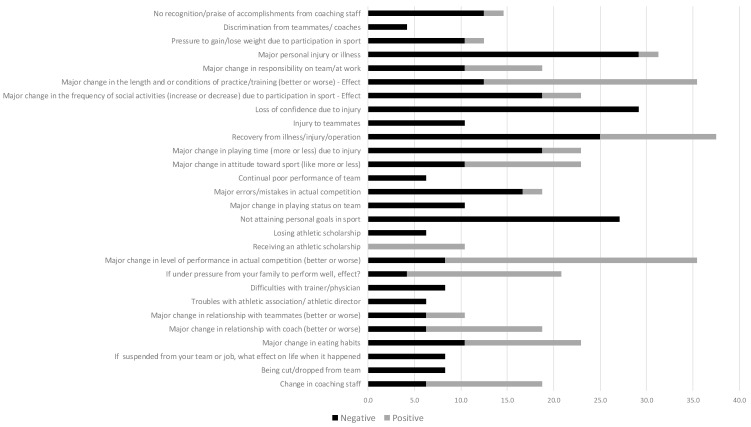
The proportions of top age-group triathletes who were affected by selected positive and negative sports-related Life Stressors in the lead up to the ITU World OD Age-group Championships.

**Figure 5:**
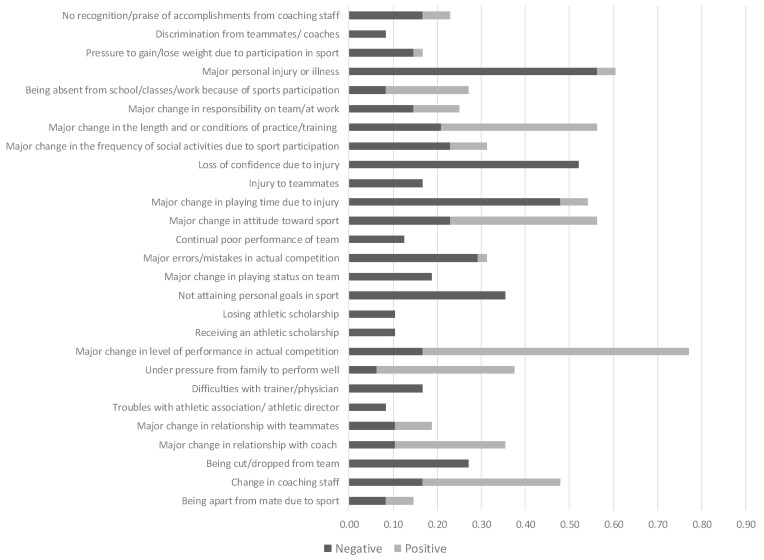
Sports-related Life Stress scores for the year leading up to the ITU World OD Age-Group Championships (arbitrary units).

Over the year leading up to the ITU World Age-Group Championships, the athletes reported themselves as having incurred Total Life stress, negative Life Stress and positive Life Stress scores of 19.1 ± 20.7 (min–max, 0–89), −19.3 ± 20.7 (0–64) and 7.3 ± 8.9 (0–36) units, respectively. Overall, 39.7 ± 33.1% of their Total Life Stress was considered to be positive and 60.4 ± 3.7% to be negative. Sports-, Relationship-, Personal- and Career-related Stress was held accountable for 42.0 ± 26.7%, 12.7 ± 18.6%, 31.3 ± 25.9% and 14.0 ± 21.1% of the athletes’ Total Life Stress, respectively. That is, most of the stress that the athletes reported via the Life Stress Questionnaire was Sports-Related Stress. The relative proportions of positive and negative stress that were reported were (i) 39.7 ± 33.1% vs. 60.4 ± 37.1% for Sport Stress; (ii) 26.8 ± 37.6% vs. 73.2 ± 37.65% for Relationship Stress; (iii) 39.6 ± 37.1% vs. 60.4 ± 37.2% for Personal Stress; and (iv) 40.1 ± 41.6% vs. 59.4 ± 41.6% for Career Stress. Sports-related Stress affected most athletes and was overwhelmingly negative when it pertained to injury, illness and/or the individual’s failure to attain his/her sporting goal(s). These findings are shown in Figure 4 and Figure 5.

## 4. Discussion

This study aimed to obtain a global overview of the training, work and Life Stress of top amateur short-distance triathletes. It also involved a preliminary assessment of the extent to which the training structure, monitoring and flexibility of such athletes may or may not be optimal. Our goal in so doing was to pinpoint potentially useful directions for research with the potential to be translated into improvement in the training practice of this athlete sub-group. We remind the reader that the survey was mainly retrospective, and that the 48 athletes whom it involved represented a small proportion of the total number of age-group athletes who were competing at the World Championships. Both issues have implications for the ability to generalize our findings beyond our sample population. We recommend, therefore, that our observations first be confirmed via a “proof of pilot” retrospective survey that itself involves a sufficiently large number of athletes for it to have adequate statistical power. The ensuing findings should then be checked via a prospective longitudinal “proof of principle” epidemiological study. Its limitations notwithstanding, however, this “pilot study” has yielded important findings.

Most of the athletes whom we surveyed were fitting their triathlon training around fixed-hour full-time work. The triathletes claimed to implement goal setting within, to periodize, to obtain feedback on and to modify their training plans when appropriate. Some evidence to support such claims was obtained. The athletes’ key focus for the season in question, in 95.8% of cases, was qualification for/performance within the ITU World Age-Group Championships [32]. However, both the timing of the placement of the Championships within the athletes’ normal training year and the way that they modified their training within the month leading up to the event suggested that the actual training that the athletes achieved was not necessarily fully commensurate with the aforementioned sporting goal. This may partly explain why Sports-related Stress accounted for the majority of the total Life Stress that the athletes reported themselves as having experienced over the year leading up to the ITU World Age-Group Championships.

Before discussing our results, it is necessary to first place the level of the age-group athletes whose data we have analyzed in context with the relevant academic literature. All of our mostly masters athletes individual and triathlon-specific personal best competition times were somewhat slower than those of the elite (but not professional) under-35-year-olds of the same sex who participated in Vleck’s prospective training diary study [14]. The latter had also been preparing for draft-legal OD competition. The OD triathlon personal best times of our males were, nonetheless, faster than those of the athletes who participated in Falk Neto et al.’s prospective study of age-group-level OD training [16]. They were also faster (at 2:13:25 ± 0:16:07 vs. 2:12:24 ± 0:02:54; in hh:mm:ss) than Aoyagi et al.’s (2021) nine younger “faster” well-trained males [6].

We mention Aoyagi’s work because it is the *only* existing report of the exercise intensities at which age-group athletes *race* the non-drafting OD competition that their training preparation is based around of which we are aware. Their 17 males raced the swim, cycle and run legs at 89.8 ± 3.7%, 91.1 ± 4.4% and 90.7 ± 5.1% of HRmax, respectively. The proportion of competition time that was spent below the aerobic threshold (HRz1), between the aerobic and the anaerobic threshold (HRz2) and above the anaerobic threshold (HRz3) was 1.5 ± 2.3%, 6.6 ± 15.0% and 91.9 ± 16.3%, respectively, for the 1.5 km swim. It was 2.8 ± 8.0%, 18.4 ± 24.0% and 78.8 ± 28.1%, respectively, for the 40 km cycle. For the 10 km run, it was 4.1 ± 10.6%, 39.9 ± 38.5% and 56.0 ± 42.1%, respectively. We note that when the athletes were split into a faster and slower group, the mean %HRmax and intensity distribution during swimming and cycling was similar in both groups. However, the faster athletes in the group spent relatively more of their running time in intensity zones z2 and z3. Thus, although our athletes, as well as being faster, also had at least four times (and in some cases double) the equivalent training/racing experience in triathlon’s component sports of Aoyagi et al.’s (younger) athletes, Aoyagi’s data give us a rough idea of what kind of *race* exercise intensity distributions the *training* data that we collected here (as shown in Table 4, Table 5, Table 6 and Table 7 and Figure 1 and Figure 2) likely related to.

This observation (despite being somewhat labored) could open up new avenues of applied research into triathlon training efficacy. There are some issues with comparing the training-related data that were obtained by the various studies, however. The most important of these is likely the fact that the various author groups did not use identical means of ascribing training intensity zones. Aoyagi et al. used laboratory-based, measurement to set zones. With Vleck’s method (Table 1), the athletes themselves set the intensity levels—albeit doing so on the basis of multiple criteria that included laboratory-derived measures. This “indirect” setting was implemented because—as was the case here—laboratory testing was not necessarily an option that was available to the athletes. Vleck’s triathletes did not record which combination(s) of the criteria for the setting of exercise intensity that are outlined in Table 1 they used. Nor did they note what proportion of this combination was derived from laboratory-based measures. In Vleck’s original study [14], however, this same method of ascribing training intensity levels (and then zones) was to some extent validated, by the athletes who took part in it, against the actual race times that they achieved over the course of the study. The same method was also part validated, in other athletes, against the results of laboratory-based incremental lactate and cardiorespiratory tests [31]. Since this is potentially highly relevant to training diary-based research into training-related maladaptation, we suggest that more detailed examination be carried out into the extent to which the method of self-ascribing training zones that was used in both Vleck’s original study and by this research (Table 1) both yield comparable results to laboratory-derived measures [33] and could be improved.

Our data may, additionally, be somewhat skewed by the fact that we asked for training duration estimations to be made to the nearest half hour. This is why we have only reported total overall training durations across and within all the sports that the athletes were doing when/where such information was obtained via direct questioning, and have not calculated total training loads. Adding up values that have been rounded up or down, from multiple questions, yields misleading numbers. Even so, however, the proportion of training time that each of our top age-groupers were spending in intensity zones z1, z2 and z3 was clearly *not* around the 75–80%, 5% and 15–20% values, respectively, that are commonly recommended, in terms of eventual performance yield, to be undertaken within each of the triathlon’s component sports [34]. The average percentage of combined swim, cycle and run training time that the entire athlete group reported itself to normally spend within z1, z2 and z3 was 53%, 33% and 14%, respectively. In no individual training block or individual triathlon discipline did the average proportion of swim, cycle and run training that the athletes spend in zone 1 exceed 56%. Falk Neto et al. [16], whose intensity zones were set via RPE and guidelines in the literature and whose training data were prospective, reported that their slower age-group athletes spent 47% of training time in z1, 25% in z2 and 28% in z3. Vleck (2010) [14,35], using the same method of ascribing training intensity level as was used in this study, but over 30 weeks of prospective longitudinal data collection, reported that their 8 faster triathletes spent 70.4%, 6.1% and 9.1% of their overall training time in their intensity levels 1–2, 3 and 4–5, respectively, i.e., in z1, z2 and z3, respectively.

It would be easy to assume, given the direction of change of the proportions of training time that were spent in each zone from the slowest to the fastest athletes in these studies, that the TID data from these three studies support Seiler’s model. This would likely be a supposition too far. We do not yet have the data to support the making of an explicit link between the proportion of training time that was spent in each intensity level by these various ability groups and their performance level (as opposed to anything else). Vleck, for example, did not compare the periodization of better vs. worse performers in the athletes who took part in her larger prospective survey. The athletes also differed on other measures that have been shown to differ among different ability levels of OD triathletes. For example, the athletes in this study possessed considerably more years of triathlon training and racing experience than Falk Neto’s similarly aged athletes (at 10.2 ± 6.2 and 9.0 ± 6.2 years, respectively, vs. 4.5 years). In an initial retrospective study, competitive experience (as well as “desire to achieve”, “stress” levels being “tense/anxious”, total mood disturbance and “can’t cope”) was shown by Vleck to differ with athlete ability level in the same group of OD athletes from which their prospective study participants were drawn [14]. It is not clear, however, seeing as they were younger, whether the athletes in Vleck’s prospective study were relatively more experienced. What is probable, given their ages, is that that they had less-demanding work commitments.

Most, but not all, the athletes in this study had full-time jobs, with fairly “normal” working hours. On average, they worked 7.6 ± 2.5 h every day. The number of times they trained per week stayed fairly constant over the year. There were some expected fluctuations from this when the triathletes were tapering or in their Off-Season (see Table 5 and Table 6). Most of the group were fitting their training around work. It seems logical that their total weekly training hours would stay fairly constant over the year, while the makeup of said training changed. As was expected, the athletes fitted the majority of their weekday training sessions in before or after what are usually considered to be normal working hours. They also did some “lunchtime” training. This is the first time that actual data, rather than anecdotal reports (as described in [14]), have been obtained regarding this point. We did not specifically ask to what extent the athletes cycled or ran to and from work. Nor—although we did obtain details of the frequency, individual and overall weekly duration of “long” and “speed” sessions in each discipline—did we inquire about how the cycling of low-, medium- and high-intensity training work was organized over an average week. This was unfortunate. It is also unfortunate that we did not inquire as to whether said training was conducted “indoors” or “outdoors”. Both issues should be followed up.

Not unexpectedly, the proportion of the athletes’ training sessions that were conducted, in each discipline, within specific time slots definitely differed between weekday- and weekend-based training (Table 4). This situation can potentially affect the extent to which recovery and adaptation might occur between the athletes’ training sessions. The degree of inherent injury risk that a given athlete was exposed to, given the timing of his/her training, could vary accordingly. For example, far more weekday than weekend based bike training was conducted in the evenings. We do not know the extent to which this training was conducted outside when daylight might be waning, or inside on a turbo trainer. Both of the latter situations might augment injury risk, but to *different extents*. Nor can we calculate the probability that, given that, it may be that the simplest reason for why these athletes are not fulfilling the recommended proportions of z1, z2 and z3 training was simply because they found it difficult to fit their training in around their other commitments. Z1 training takes longer to complete. The possibility that (e.g., employment-related) limitations in their training time leads these athletes to do high amounts of higher-intensity training than they might otherwise have chosen to do was raised by McCormick [27]. Although the sample numbers were too small for the authors to be sure, McCormick’s point appears to have been supported by Falk Neto’s unpublished data [36]. These age-groupers were doing at least some of their training in organized group/club sessions, which are unlikely to have included much z1 training.

Certainly, club-related socialization may have had some positive repercussions in terms of the athletes’ general mental and social health. Most of our subjects were enrolled in single-sport- rather than in triathlon-specific clubs, however. They were not, therefore, necessarily receiving training that took what they were doing in the other triathlon disciplines into account while in that environment (Table 3). Indeed, in answer to the question “if you are coached (other than self-coached), and your training is geared towards triathlon, do you have the same coach overseeing all three disciplines”, only 27.1% of the group answered “always.” Even so, some level of training periodization among the Endurance-Base, Pre-competition, Taper and Competition phases of the training year, as well as in the Off-Season, was demonstrated in the athletes’ replies to the online survey. They achieved this periodization across training phases within each individual triathlon discipline. They also periodized their overall swim, bike and run training (as shown in Table 5, Table 6 and Table 7 and Figure 1 and Figure 2). We saw similar results regarding the periodization that occurred both within and across training phases and disciplines when we extended the number of athletes in the training-related dataset up to 124. We did so by adding the data for those athletes for whom up to 3 out of 45 training intensity-related replies were missing into the existing dataset [31]. Nonetheless, our data that specifically related to how the athletes trained within the one-month lead up to the World Age-Group Championships indicate that the extent to which these age-groupers modified their training intensity distribution, racing load *and inter-session recovery* in the light of external factors (Figure 3) may not always have been ideal. We observed that, as the athletes were representing multiple countries (and time zones of origin), “Worlds” did not necessarily fall within the competition period of each individual athlete’s training year. It was certainly unclear from the data that we obtained—which examined how their training for that month was modified from what was normal for them for the macro-cycle in question—to what extent the athletes took this issue into account.

The level of feedback that the athletes were receiving on their training in relation to their perceived ability also supports the premise that their training adjustment process could, nonetheless, perhaps be improved. The data that we obtained regarding the extent to which these age-groupers acted in response to the failure of that training (i.e., injury, illness or unexplained performance decrement) support this assertion. So, too, does the extent to which such failure occurred, as exemplified by the injury prevalence values that we obtained. We further highlight the fact that 40% of the athletes in this study self-identified themselves as allergy sufferers. Similar observations were made for the larger 602-athlete sample from which this group was drawn [31]. Although we could find no comparative data for amateur triathletes to compare this to in the academic literature, Teixeira et al. [37] observed allergy symptoms in 54.2% of a mixed sample of 59 elite triathletes and runners. This observation may point to an adverse, potentially training-related, immune status in at least some amateur triathletes. The results that are reported here in age-group triathletes are clearly worth following up on with at least the Allergy Questionnaire for Athletes [37], if not more [38].

We consider it highly noteworthy, moreover, that the majority of the Life Stress that the athletes reported was Sports-related Stress. Outstanding personal achievement and a major change in performance in actual competition, recovery from illness/injury and a major change in academic/work activity were the triathletes’ main sources of Life Stress over the year leading up to the World Championships. The LESCA does not directly enquire about stress accruing from difficulties in maintaining what the athlete perceives to be the appropriate life work/sports balance. Nonetheless, the fact that a major change in work/academic activity was a source of stress could indicate work/sports balance to be an important issue. We note that the issues that were identified as directly sports-related were the ones that caused the most negative stress (as illustrated in Figure 4 and Figure 5). They included not attaining personal goals in sports, major personal injury or illness, a major change in playing (i.e., training) time due to injury, recovery from injury/illness/operation and loss of confidence due to injury, as well as a lack of recognition of the athlete’s accomplishments from coaching staff. As we only assessed 48 athletes here, there were not enough of such injury/illness data to bother assessing what they were linked to. Given the level of detail that the athletes provided on the etiology of any injury/illness that they sustained for that month (far more so than for the other time periods that we assessed), we reserve such an analysis for our larger 602-athlete sample. It is perhaps a pity, however, that the LESCA data were (also) not obtained over the same one-month time frame. This will limit the extent to which they can be linked to injury in particular.

## 5. Conclusions and Future Directions

We feel that the scheduling, completion, TID distribution and possible efficacy of this group’s training may be linked to the “work/life/sport“ scheduling conflicts that are associated with being “part-timers.” The minimal sociocultural data that exist for triathletes thus far have suggested them to be generally well-educated individuals who also excel in their business lives. They have also, however, been reported to exhibit less (sports-related) harm avoidance behavior than their single-sport counterparts [39]. We recommend that systematic examination of both the benefits and barriers that are experienced by age-group triathletes in combining their sports, academic and/or professional careers be carried out. We also recommend that the impact that such issues can have on the physical and mental health and well-being of such athletes be assessed [40].

This wide-ranging study has highlighted just how much research into training and adaptation in age-group triathletes still needs to be conducted before its results can easily be translated into improvement in training practice. Given that it was retrospective and involved a small percentage of the total number of athletes who took part in the event, we firstly need to examine the extent to which the findings of this study may be extrapolated to the wider subject population. We saw that the TID of these amateur triathletes is not apparently what has been considered to be best practice for *single-discipline* endurance sports. But, the TID values that we calculated were based on retrospective data. They also do not account for the athletes’ weight training, for their training in other sports or for their off-training activity. Moreover, the triathlon has repeatedly been acknowledged to be more than the sum of its single-sport counterparts. Its cycle training and run training have certainly already been shown to have synergistic, cumulative effects [18,41]. Perhaps the most important question here, therefore, is not so much the holistic one of “is classic polarized training ideal for triathlon?” As Sperlich et al. [8] recently pointed out: ”it may be questionable if a general best-practice or “optimal” TID exists at all, and if so, the replication of TID will not be feasible in the long run”. The far more pressing question to be addressed is surely a far more individualistic one. It is “what is the best training practice that a given age-group athlete can feasibly achieve, given his/her work and other commitments?” This study revealed significant differences between weekday- and weekend-based training in the timing of, and therefore, in the recovery/training adaptation periods between, successive training sessions. As triathlon coaches, we make decisions on the timing and order of our athletes’ successive swim, cycle, bike and weight training sessions that are based more on our personal experience than on any available scientific evidence. Swimming, for example, is generally considered to have less impact than running. It is often chosen over running, for that reason, to follow a high-intensity turbo training session. Examination, in top age-groupers, of the order in which they cycle high-, medium- and low-intensity work across the (four,- including weight training) triathlon disciplines may prove useful. So, too, may investigation into how top amateurs adjust this type of periodization in view of their work demands. The dual careers literature may prove to be helpful to investigators in this regard.

The second logical follow-up to these studies is an investigation of how exactly their “within” and “outside” training stressors may have influenced maladaptation in the larger retrospective study of 602 triathletes—for which sufficient injury and illness data appear to be available to do this—of which they formed a part. How these influenced training needs to be followed up. So, too, do the possible explanations of why said training diverged from either accepted or the individual athlete’s norms. Far more athletes reported their inter-session recovery to be *less* (as opposed to the same as or higher) than normal during the month-long lead up to the World Championships, for example. They did so, even though they had confirmed that the World Championships were the focus of their entire training year. Did the same finding occur in the larger sample of 602 athletes? If so, why? What might have influenced this departure from accepted training wisdom? What research questions need to be addressed for us to be able to use the answers to improve training practice?

Of additional interest are the average weekly number of (swim, cycle, run and resistance) training sessions that the triathletes did, regardless of when the athletes were in their training year, and how these data relate to the most recently published updated exercise training guidelines for masters athletes [15]. “A combination of exercise stresses (endurance, sprint, and strength) is likely required to optimally maintain physical capacity into older age… Athletes should do only one to two threshold or high-intensity training sessions per week, interspersed with two to four long slow distance sessions per week, depending on their training history. They should also factor in one or two strength training sessions per week.” Investigation of how variable “normal” athlete training within each of the various triathlon age-groups is, and how such “normal” training varies from one age-group to another, in tandem with examination of how this is reflected by decreases in actual performance, would be the logical follow-up to this observation. For such research to be successful, far larger sample sizes are needed than we utilized in this first exploratory study. Research into how the risk factors that have already specifically been linked to the occurrence of overuse injury in triathletes [2,20,21,29,30] differ across age-groups, and change across the athlete’s life span, is also likely to yield results that could have important implications for the improvement of training practice in age-group triathletes.

## Figures and Tables

**Table 1:** The explanations of training intensity levels that were provided within the Vleck (2013) training and injury survey *.

Training Intensity Level	
1	Intensity: low, well under lactate threshold (LT), 60–70% HRmax. RPE easy, 1–3. Rest: little or none. Energy source: fats. Examples: Easy workouts/recovery sets, warm up/cool down.
2	Intensity: 1–2 mM below LT, 70–75% HRmax. Easy to moderate. RPE 2–4. Rests 05–15 s. Energy source: fats with marginal CHO. Examples: Swim long intervals with very short rests (3 × 800 m w/15 s, 5 × 400 m w/10 s, 12 × 200 m w/5 s), continuous swims. Bike: 1.5–3 h of continuous riding. Long, easy 1–2 h runs.
3	Level of exertion: moderate. RPE 4–6. Rest 10–30 s. Energy source: CHO with some fat. Examples: Swim long intervals with short rest (5 × 500 m w/20 s, 3 × 800 m w/20 s, 8 × 250 m w/15 s, 20 × 100 m w/10 s), continuous (1500–3000 m) swims. Bike: 30–90 min continuous riding. Straight brick sessions (bike: run). Short rest intervals, e.g., 2 × 20 min w/2 min, 3 × 12 min w/1 min, 4 × 8 min w/30 s. Run: 20–30 min continuous. Straight brick running.
4	Level of effort: moderate to hard maximal steady-state training. RPE 6–8. Rest 15–45 s. Energy source: CHO with marginal fat. Examples: Swim moderate intervals with moderate rest (12 × 100 m). Volume range usually 1000–2000 m. Bike brick intervals; moderate length intervals with moderate rest (6 × 5 min w/2 min, 3 × 8 min w/4 min, 8 × 3 min w/1 min); time trials (10–20 miles) and races. Run brick intervals; moderate length intervals with moderate rests (e.g., 5 × 3 min w/1.5 min, 4 × 4 min w/2 min, 3 × 8 min w/4 min, etc.), or races.
5	Level of effort: hard-maximal and near maximal quality training, speech impossible. RPE 9–10. Energy source: CHO. Work to rest ratio 1:2 to 1:4. Examples: Swim short reps with long rests (e.g., 10 × 50 m odd easy, even-FAST; 6 × 75 m building by 25′s w/1 min, 12 × 25 m sprints leaving on 30 s), at or near maximum pace. Volume range usually 400–1000 m. Bike hill repeats using hills that take 2–3 min to climb at a fast sustained pace; or short intervals with long rests (8 × 1 min w/2 min, 12× 1.5 min w/1 min etc.). Run hill repeats (200–400 m length) or repeats with long rest (e.g., 8 × 400 w/2 min, 5 × 800 w/4 min or 12 × 200 w/1 min).

Key: CHO, carbohydrate; %HRmax, percentage of (sport-specific) maximum heart rate; LT, lactate threshold; m, metres; max, maximum; min, minutes; Mm, millimolar; reps, repetitions; RPE, rating of perceived exertion (10-point Borg scale); s, seconds; w/, with rest/recovery of. * More details of what the various intensity levels equated to were given in Vleck (2010)—where they were termed “L1, L2, L3 easy (extensive anaerobic threshold), L3 hard or intensive anaerobic threshold (i.e., 40 km bike and 5–10 km run race intensity), and Level 4 (intensity very high, 2–6 mM above LT)”.

**Table 2:** Age-groups and gender distribution of the study subjects.

Age-Group	20–24	25–29	30–34	35–39	40–44	45–49	50–54	55–59	60–64	65–69
N (of whom M)	2 (1)	5 (2)	8 (3)	5 (3)	3 (2)	4 (1)	7 (3)	6 (3)	4 (2)	4 (1)

Key: N, number; M, males.

**Table 3:** Coaching and its specificity—% of athletes *.

Type of Coaching	Swim	Bike	Run
None	4.2	14.6	10.4
Self-coached	16.7	31.3	25.0
Club-based coaching	39.6	18.8	29.2
Internet-based coaching	6.3	4.2	4.2
Other type of coaching	33.3	-	-
Triathlon specific	37.5	37.5	35.4
Single-sport specific	18.8	6.3	8.3

* The level of coaching specificity that the athletes received was shown to be significantly associated, at the *p* < 0.01 level, with which of the three triathlon disciplines it was being provided within, by the Chi-squared test. Key: -, no reply.

**Table 4:** The scheduling of “working week” vs. weekend-based training in top short-distance amateur triathletes (mean ± SD).

During the Week						
		Swim (S)	Bike (B)	Run (R)	Weights (W)	Other (O)
Sessions (over 5 days) ^a^		2.7 ± 1.0 ***	2.1 ± 1.3 ***	2.2 ± 1.1 ***	1.0 ± 1.3 ***	0.3 ± 0.6 *
As % of within week total	Before 9 a.m.	29.8 ± 38.9	28.7 ± 41.3	22.0 ± 38.4	16.7 ± 35.4	16.7 ± 35.4
	9 a.m.–12 p.m.	14.3 ± 32.0 ***	16.9 ± 32.4 ***	12.0 ± 29.9	25.9 ± 43.4	25.9 ± 43.4
	12 p.m.–3 p.m.	6.2 ± 15.7	15.2 ± 28.1	12.0 ± 29.9	3.7 ± 11.1	3.7 ± 11.1
	3 p.m.–6 p.m.	21.4 ± 31.9 ***	16.9 ± 34.0 *	30.7 ± 46.1	15.0 ± 33.7	15.0 ± 33.7
	6 p.m.–10 p.m.	28.4 ± 34.4	27.7 ± 42.5 ***	24.0 ± 41.1	37.0 ± 48.4	37.0 ± 48.4
**At Weekends**						
		**Swim (S)**	**Bike (B)**	**Run (R)**	**Weights (W)**	**Other (O)**
Sessions (over 2 days) ^b^		0.4 ± 0.6	1.3 ± 0.7	1.3 ± 0.8	0.1 ± 0.3	0.0 ± 0.3
as % of weekly total		11.3 ± 19.8	44.6 ± 26.0	36.9 ± 19.3	9.3 ± 22.9	10.0 ± 31.6
As % of weekend total	Before 9 a.m.	48.9 ± 50.2	34.4 ± 46.2	28.4 ± 43.9	0	0
	9 a.m.–12 p.m.	17.8 ± 37.5	51.5 ± 48.3	50.9 ± 48.2	0	0
	12 p.m.–3 p.m.	26.7 ± 45.8	8.5 ± 24.0	14.9 ± 31.7	60.0 ± 54.8	0
	3 p.m.–6 p.m.	6.7 ± 25.8	3.3 ± 16.5	3.5 ± 12.9	40.0 ± 54.8	100
	6 p.m.–10 p.m.	0	0	2.3 ± 15.2	0	0

Note (statistics): Wilcoxon test (week vs. weekends): ***, *p* < 0.001; *, *p* < 0.05. Friedman’s test (swim vs. bike vs. run vs. weights vs. other): ^a^, *X*^2^(4) = 103.084, *p* < 0.001, N = 47; ^b^, *X*^2^(4) = 131.649, *p* < 0.001, N = 47. Post hoc tests, after Bonferroni correction, significant at *p* < 0.001, during the week, for S-W, S-O, B-W, B-O, R-W, R-O, and during the weekend, for S-B, S-R, B-W, B-O, R-W, R-O.

**Table 6:** Swim, bike and run training weekly session frequency and total time (h): details (mean ± SD).

Variable			EB	PC	C	T	Off
Swim-Bike Transition (T1)		Frequency	1.2 ± 0.7	1.4 ± 0.7	1.5 ± 0.8	1.3 ± 0.6	1.1 ± 0.4
		Total time (h)	1.1 ± 0.3	1.5 ± 0.9	1.6 ± 0.9	1.4 ± 0.8	1.0 ± 0.0
Long Bike (LB)		Frequency	2.3 ± 0.7	2.2 ± 0.6	2.1 ± 0.7	1.3 ± 0.4	1.7 ± 0.8
		Total time (h)	7.1 ± 3.0	6.6 ± 3.2	5.3 ± 2.7	2.8 ± 2.2	4.5 ± 3.1
Hill Reps Bike (HRB)		Frequency	1.5 ± 0.6	1.7 ± 0.6	1.7 ± 0.6	1.2 ± 0.4	1.2 ± 0.4
		Total time (h)	2.0 ± 1.3	2.6 ± 1.3	2.5 ± 1.4	1.4 ± 0.9	1.3 ± 0.7
Speed Work Bike (SWB)	Field	Frequency	1.3 ± 0.5	1.8 ± 0.6	2.0 ± 0.7	1.7 ± 0.6	1.1 ± 0.4
	T/T	Frequency	1.8 ± 0.7	1.6 ± 0.6	1.4 ± 0.5	1.3 ± 0.5	1.4 ± 0.6
	Both	Total time (h)	2.5 ± 1.5	3.5 ± 1.4	3.7 ± 1.6	2.7 ± 1.2	1.8 ± 1.2
Bike–Run Transition (T2)		Frequency	1.4 ± 0.6	1.8 ± 0.9	1.9 ± 0.8	1.7 ± 0.9	1.0 ± 0.2
		Total time (h)	1.6 ± 1.1	2.2 ± 1.5	2.5 ± 1.4	1.9 ± 1.1	1.1 ± 0.4
Other Bike (OB)		Frequency	1.6 ± 0.7	1.7 ± 1.0	1.7 ± 1.0	1.6 ± 0.7	1.9 ± 0.9
		Total time (h)	3.0 ± 2.7	2.8 ± 2.6	2.8 ± 2.6	2.4 ± 2.1	2.9 ± 2.3
Long Run (LR)		Frequency	2.1 ± 0.5	2.0 ± 0.6	1.8 ± 0.8	1.3 ± 0.5	1.8 ± 1.0
		Total time (h)	4.7 ± 2.0	4.1 ± 2.3	3.6 ± 3.0	2.0 ± 1.2	3.3 ± 1.9
Hill Reps Run (HRR)		Frequency	1.5 ± 0.5	1.5 ± 0.6	1.4 ± 0.5	1.1 ± 0.3	1.2 ± 0.5
		Total time (h)	1.8 ± 1.1	2.0 ± 1.1	1.7 ± 1.0	1.3 ± 0.8	1.5 ± 1.0
Speed Work Run (SWR)		Frequency	1.9 ± 0.7	2.2 ± 0.6	2.3 ± 0.6	1.9 ± 0.6	1.5 ± 0.6
		Total time (h)	2.5 ± 1.3	3.2 ± 1.5	3.5 ± 1.5	2.4 ± 1.0	1.6 ± 0.9
Other Run (OR)		Frequency	2.0 ± 1.2	1.9 ± 1.2	1.8 ± 1.1	1.8 ± 1.0	2.1 ± 1.2
		Total time (h)	3.3 ± 2.7	3.2 ± 2.5	3.1 ± 2.5	2.7 ± 2.2	3.0 ± 2.4

Key: EB, Endurance Base; PC, Pre-Competition; C, Competition; T, Taper; Off, Off-Season; T/T, turbo trainer; total time (in h, not hh:mm, because the question only asked for data to the nearest half hour).

**Table 7:** Weekly self-reported usual training time in three intensity zones and five types of training blocks (mean hh:mm ± SD).

	Intensity Level (Zone in Brackets)			ANOVA		Post Hoc Test ^a^		
	L1–2 (z1)	L3 (z2)	L4–5 (z3)	*F*(2,143)	Sig.	z1–z2	z1–z3	z2–z3
Endurance-Base								
Swim	1:30:37 ± 1:16:40	1:00:37 ± 0:44:24	0:30:37 ± 0:36:52	14.073	<0.001	0.027	<0.001	0.027
Bike	2:43:07 ± 2:05:45	1:28:07 ± 0:55:50	0:46:52 ± 0:54:58	22.784	<0.001	<0.001	<0.001	ns
Run	2:06:15 ± 1:11:53	1:05:37 ± 0:57:36	0:31:15 ± 0:29:39	14.943	<0.001	<0.001	<0.001	ns
Total	6:19:59 ± 4:34:18	3:34:21 ± 2:37:50	1:48:44 ± 2:01:29	27.969	<0.001	<0.001	<0.001	ns
**Pre-Competition**								
Swim	1:08:45 ± 0:53:56	1:08:07 ± 0:36:59	0:55:37 ± 0:42:25	1.299	ns	ns	ns	ns
Bike	2:21:52 ± 1:56:33	1:51:52 ± 1:03:56	1:13:07 ± 0:49:28	8.502	<0.001	ns	<0.001	ns
Run	1:46:15 ± 1:03:24	1:11:15 ± 0:52:44	0:50:00 ± 0:35:43	6.136	0.003	0.009	0.009	ns
Total	5:16:52 ± 3:53:53	4:11:14 ± 2:33:39	2:58:44 ± 2:07:36	7.354	0.001	ns	0.001	ns
**Competition**								
Swim	0:54:22 ± 0:47:47	1:08:07 ± 0:39:58	0:55:37 ± 0:42:25	1.465	ns	ns	ns	ns
Bike	2:05:37 ± 1:54:13	1:53:07 ± 1:10:37	1:21:52 ± 1:03:38	3.311	0.039	ns	0.041	ns
Run	1:30:00 ± 0:59:59	1:07:30 ± 0:48:08	0:54:22 ± 0:42:16	2.951	ns	ns	ns	ns
Total	4:29:59 ± 3:41:59	4:08:44 ± 2:38:43	3:11:51 ± 2:28:19	2.494	ns	ns	ns	ns
**Taper**								
Swim	0:57:30 ± 0:50:35	0:48:45 ± 0:34:14	0:35:37 ± 0:33:42	3.586	0.030	ns	0.026	ns
Bike	1:46:15 ± 1:50:31	1:16:52 ± 1:00:36	0:43:44 ± 0:38:37	8.101	<0.001	ns	<0.001	ns
Run	1:14:22 ± 0:55:20	0:43:07 ± 0:28:57	0:28:45 ± 0:34:25	9.886	<0.001	0.001	0.001	ns
Total	3:58:07 ± 3:36:26	2:05:37 ± 2:03:47	1:48:06 ± 1:46:44	9.934	<0.001	0.026	<0.001	ns
**Off-Season**								
Swim	1:22:30 ± 1:20:48	0:44:22 ± 0:39:37	0:20:37 ± 0:42:29	15.183	<0.001	0.001	<0.001	ns
Bike	2:14:59 ± 1:52:35	0:55:37 ± 0:59:40	0:16:52 ± 0:27:36	30.713	<0.001	<0.001	<0.001	ns
Run	1:51:52 ± 1:08:16	0:48:44 ± 0:50:30	0:19:22 ± 0:31:51	19.566	<0.001	<0.001	<0.001	ns
Total	5:29:21 ± 4:21:39	2:28:43 ± 2:29:47	0:56:51 ± 1:41:56	43.692	<0.001	<0.001	<0.001	ns

Key: ^a^, with Bonferroni correction; L1–2, intensity levels 1 and 2; L3–4, intensity levels 3 and 4; L3, intensity level 3; z1, zone 1; z2, zone 2; z3, zone 3; Sig., significant; ns, non-significant.

**Table 8:** Proportion of the athletes who went into the race with pre-existing morbidity factors, injury and/or illness.

Percentage Affected (with % Who Were Unsure in Brackets)			
Family History of CV Disease	22.9 (6.3)	Asthma	14.6 (2.1)
Current CV problems	10.4	Ex-induced asthma	14.6 (2.1)
Ever fainted, blacked out or had dizzy spells	27.1	Hay fever	22.9
Ever experienced loss of consciousness or fainting with exercise	8.3	Allergies	39.6 (2.1)
Have/had serious illness or condition that could affect current ability to exercise			18.8

Key: CV, cardiovascular; Ex-induced, exercise induced.

## Data Availability

The dataset contains private medical information. It is not publicly available.
